# Incidence of Community-Acquired Lower Respiratory Tract Infections and Pneumonia among Older Adults in the United Kingdom: A Population-Based Study

**DOI:** 10.1371/journal.pone.0075131

**Published:** 2013-09-11

**Authors:** Elizabeth R. C. Millett, Jennifer K. Quint, Liam Smeeth, Rhian M. Daniel, Sara L. Thomas

**Affiliations:** Faculty of Epidemiology and Population Health, London School of Hygiene and Tropical Medicine, London, United Kingdom; Charité, Campus Benjamin Franklin, Germany

## Abstract

Community-acquired lower respiratory tract infections (LRTI) and pneumonia (CAP) are common causes of morbidity and mortality among those aged ≥65 years; a growing population in many countries. Detailed incidence estimates for these infections among older adults in the United Kingdom (UK) are lacking. We used electronic general practice records from the Clinical Practice Research Data link, linked to Hospital Episode Statistics inpatient data, to estimate incidence of community-acquired LRTI and CAP among UK older adults between April 1997-March 2011, by age, sex, region and deprivation quintile. Levels of antibiotic prescribing were also assessed. LRTI incidence increased with fluctuations over time, was higher in men than women aged ≥70 and increased with age from 92.21 episodes/1000 person-years (65-69 years) to 187.91/1000 (85-89 years). CAP incidence increased more markedly with age, from 2.81 to 21.81 episodes/1000 person-years respectively, and was higher among men. For both infection groups, increases over time were attenuated after age-standardisation, indicating that these rises were largely due to population aging. Rates among those in the most deprived quintile were around 70% higher than the least deprived and were generally higher in the North of England. GP antibiotic prescribing rates were high for LRTI but lower for CAP (mostly due to immediate hospitalisation). This is the first study to provide long-term detailed incidence estimates of community-acquired LRTI and CAP in UK older individuals, taking person-time at risk into account. The summary incidence commonly presented for the ≥65 age group considerably underestimates LRTI/CAP rates, particularly among older individuals within this group. Our methodology and findings are likely to be highly relevant to health planners and researchers in other countries with aging populations.

## Introduction

Pneumonia and lower respiratory tract infections (LRTI) are major causes of morbidity and mortality among those aged 65 years and over in the UK and other European countries [1–3]. The UK’s population is aging; recent estimates suggest that in 2035, 23% of the UK will be aged ≥65 years and 5% will be ≥85, compared to 17% and 2% respectively in 2010 [4]. The ‘oldest old’ (≥85 years) are at particularly high risk of infections due to co-morbidities and waning immune function. Community-acquired pneumonia (CAP) in older individuals is a particular concern, as it can aggravate underlying co-morbidities and have serious consequences [5]. Thus, the need has been highlighted for new population-based studies of the incidence of these infections among older adults in different European locations [6].

There are few longitudinal studies on the burden of these infections specifically amongst older adults in the UK. This is a disparate group, including people working full-time and those that require round-the-clock care. Available incidence estimates vary, partly due to different age categorisations and methods used [1,7,8], and community- and hospital-acquired infections are rarely differentiated. Existing studies of regional and socio-economic variations in incidence have not age-stratified further after 65 years. Therefore there is a paucity of information for this important and growing subsection of the population; it is essential that their LRTI burden is better understood to enable planning and provision of health care. The extent of antibiotic prescribing in general practice for both LRTI and CAP among older adults also needs ongoing assessment.

The aim of this study was to describe the incidence of community-acquired LRTI and CAP among individuals aged ≥65 years between April 1997 and March 2011, using linked electronic health records from primary and secondary care. We describe how the incidence of these common infections varied over time by age, sex, region and socioeconomic deprivation and the extent of antibiotic prescribing in this group.

## Methods

### Data sources

The Clinical Practice Research Datalink (CPRD, formerly known as GPRD) is a large UK-based electronic database of primary care records which currently includes around 8% of the UK population. The age, sex and regional distribution of patients from contributing practices are representative of the UK overall [9]. Anonymised patient-level information including diagnoses (coded using Read codes), referrals to specialist care, prescriptions, hospitalisations, demographic and lifestyle details are included. Data from a practice are only used for research after they have met a series of quality checks and have been deemed ‘up to standard’ by CPRD.

Over 50% of English practices that contribute to CPRD consent to linkage of their patients’ records to Hospital Episode Statistics (HES) data. These contain information on all NHS inpatient hospitalisations in England since 1997, with diagnoses coded using ICD-10. Hospitalisations include one or more ‘episodes’, each denoting a period of consultant care.

### Study population and follow-up time

Patients aged ≥65 years between 1^st^ April 1997 and 31^st^ March 2011 were eligible for inclusion. Follow-up began at the latest of the study start date, patients’ 65^th^ birthday, the date CPRD deemed the practice ‘up to standard’ or 28 weeks after patients’ registration (to exclude reports of historical illness that are often recorded when a patient first joins a practice; the 28-week period was chosen after analyses based on existing methods) [10]. Follow-up ended at the earliest of the study end date, death, transfer out of the CPRD or the practices’ last data collection date. Patients who contributed at least one day of follow-up were included in the study.

### Codes used to define LRTI, hospitalisations and antibiotics

Read and ICD-10 code lists for LRTI and within this list, pneumonia were developed by three clinical epidemiologists, including a consultant respiratory physician and a GP. An estimated 50-70% of chronic obstructive pulmonary disease (COPD) exacerbations are due to LRTI [11]; COPD exacerbation codes that did not mention infection were also identified for sensitivity analyses.

We examined evidence of hospitalisations to distinguish between potentially hospital-acquired and community-acquired infections. In the CPRD data, we identified hospitalisation codes and relevant fields indicating hospitalisation in the consultation, referral and clinical files (code lists available on request).

Antibiotic therapy codes were identified and categorised by their British National Formulary (BNF) subchapter, excluding antituberculosis and antileprotic drugs [12–15].

### LRTI/pneumonia illness-episode structure

Records containing LRTI or pneumonia codes were identified in both CPRD and HES. In order to accommodate multiple consultations for one illness, CPRD or primary HES LRTI/pneumonia records within 28 days of each other were regarded as part of the same illness-episode. The first record was deemed the index date and the illness-episode finished 28 days after its last LRTI code. Within HES, only LRTI/pneumonia codes recorded as the primary code of the first episode of a hospitalisation (the condition the patient was admitted for) were used when defining the index date, to avoid including hospital-acquired infections.

As pneumonia formed a subset of LRTI, pneumonia illness-episodes could start on the same date as an LRTI episode (if the patient initially presented with pneumonia) or at some point within an LRTI episode (if an LRTI had worsened).

### Defining community-acquired illness

Incident cases of LRTI were regarded as hospital-acquired if in the previous 14 days the patient had been discharged from hospital (using HES records for any illness) or there was a CPRD hospital code (using the unlinked data) [14,16–19].

### Other variables

We used the ‘financial year’ definition of April–March to ensure the winter peak of LRTI was not split across two years. Age was grouped in five-year bands from 65 to 89 years, then as ≥90 years. English regions were defined by Strategic Health Authority (SHA). Index of Multiple Deprivation (IMD) quintile (2007) was available at Office for National Statistics (ONS) small area level (100 houses) for >50% of CPRD patients. The IMD is calculated using seven domains: income, employment, health deprivation and disability, education, skills and training, barriers to housing and services, crime and living environment.

### Analyses

At their simplest, incidence rates are calculated by dividing the number of new episodes of illness by the person-time at risk. Patients were considered not at risk of a community-acquired LRTI during an LRTI illness-episode (whether community or hospital-acquired), during a HES hospitalisation or for the 14 days after any HES hospitalisation or CPRD hospital code. This person-time was excluded from the denominator when calculating incidence.

Incidence rates were calculated by year, sex, age, region of England and IMD quintile. Poisson regression with random effects was used to account for multiple illness-episodes per person. Incidence rates were directly age-standardised using the ONS mid-year UK population estimates from 2004, and 95% confidence intervals (CI) were calculated.

We identified how many patients had been prescribed an antibiotic on the illness index date, and the type of antibiotic prescribed. Among those without a prescription on the index date, we calculated the percentage that were hospitalised or died as possible reasons for not having received a GP prescription. For remaining patients, analyses were repeated sequentially for the week after, and then two to four weeks after the index date. Finally, records in the week before the index date were examined to assess how many patients had received an antibiotic prescription in this time.

Statistical analysis was conducted using Stata version 11.2 and Microsoft Excel.

### Ethics information

All data were anonymised prior to receipt by the authors. Ethics approval for the study was given by the Independent Scientific and Advisory Committee (of CPRD), and the London School of Hygiene and Tropical Medicine Ethics Committee.

## Results

The study population comprised 1,534,443 patients from 625 practices across the UK (Table 1). Over half the participants were aged 65-69 at the start of the study and 56% were female. Median period of patient follow-up was 5.1 years (interquartile range (IQR): 2.3-9.2). HES-linked information was available for 59.7% of patients, whose characteristics were largely similar to those of the whole cohort (Table 1).

**Table 1:** Characteristics of the study population, for all patients and for patients with HES-linked data.

	**Entire study population**		**HES-linked patients**
	n (%)		n (%)
**Number of patients**	1534443		916128 (59.7)
**Median years follow-up (IQR)**	5.1 (IQR:2.3-9.2)		5.3 (IQR:2.3-9.6)
**Sex**			
**Male**	672858 (43.9)		402474 (43.9)
**Female**	861585 (56.1)		513654 (56.1)
**Age at start of follow-up**			
**65-69**	819333 (53.4)		491205 (53.6)
**70-74**	237349 (15.5)		140743 (15.4)
**75-79**	197400 (12.9)		117298 (12.8)
**80-84**	142077 (9.3)		84533 (9.2)
**85-89**	89292 (5.8)		53559 (5.8)
**90+**	48992 (3.2)		28790 (3.1)
**Region practice is based^a^**		England only %	
**North East**	29432 (1.9)	2.4	20615 (2.3)
**North West**	178011 (11.6)	14.5	145665 (15.9)
**Yorkshire & The Humber**	71012 (4.6)	5.8	46482 (5.1)
**East Midlands**	60824 (4.0)	5.0	32744 (3.6)
**West Midlands**	130162 (8.5)	10.6	106627 (11.6)
**East of England**	150977 (9.8)	12.3	115749 (12.6)
**South West**	144749 (9.4)	11.8	128592 (14.0)
**South Central**	165094 (10.8)	13.5	110107 (12.0)
**London**	155219 (10.1)	12.7	106431 (11.6)
**South East Coast**	139681 (9.1)	11.4	103116 (11.3)
**Northern Ireland**	43633 (2.8)		N/A
**Scotland**	121428 (7.9)		N/A
**Wales**	144221 (9.4)		N/A
**Index of Multiple Deprivation (IMD) quintiles relative to country as a whole**			
**Unavailable**	712107 (46.4)		99150 (10.8)
**0 (least deprived)**	190874 (23.2)		189466 (23.2)
**1**	204589 (24.9)		203345 (24.9)
**2**	171462 (20.9)		170527 (20.9)
**3**	150185 (18.3)		149164 (18.3)
**4 (most deprived)**	105226 (12.8)		104476 (12.8)

^a^ Hospital Episode Statistics (HES) data available for England only

### Incidence by age and sex

**Figure 1:**
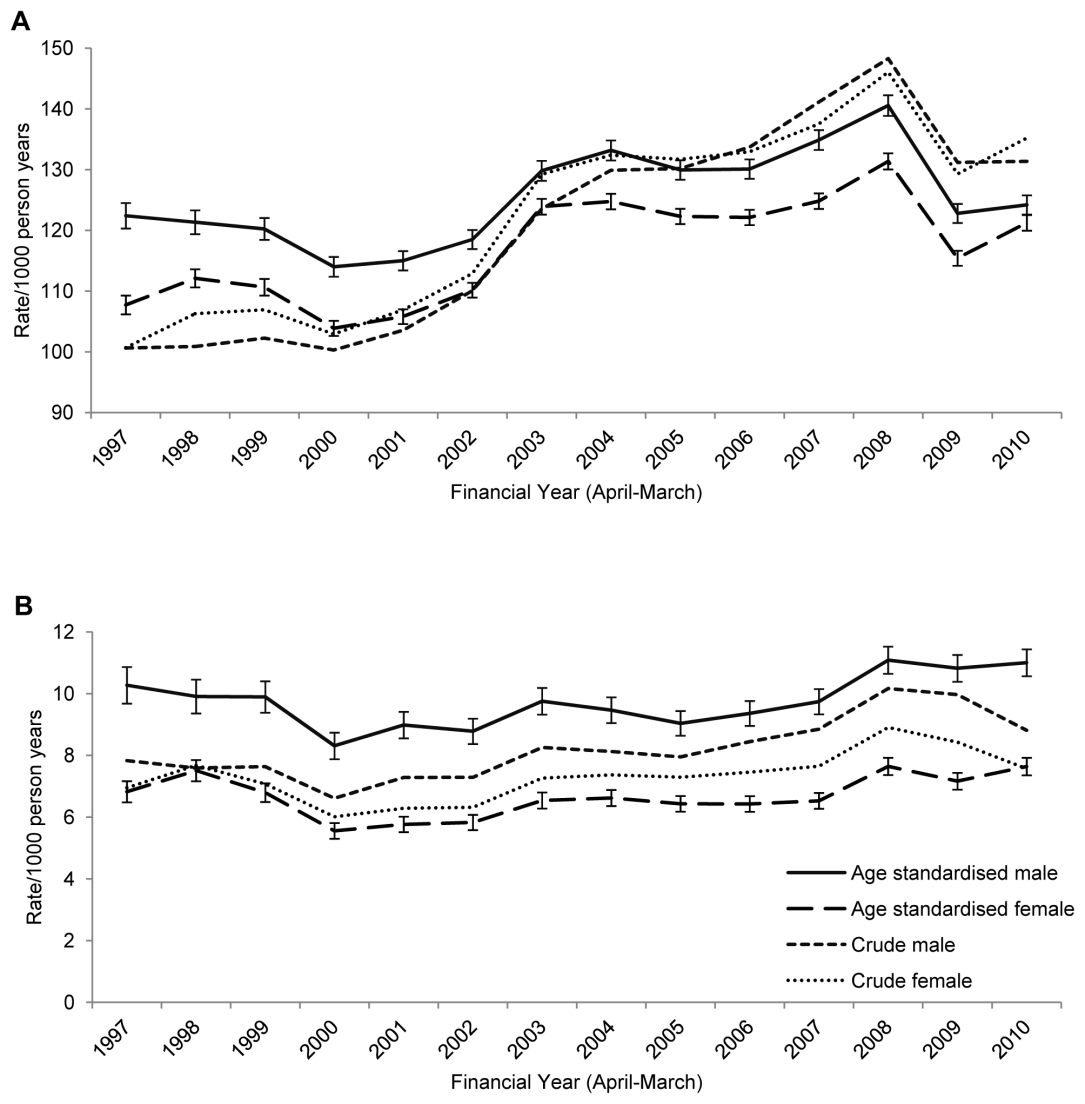
Incidence of LRTI and CAP by sex over time. Crude and age-standardised incidence of a) LRTI and b) CAP by sex over time. Standardised to UK population, mid-year 2004.

Over the 14-year study period, 974,121 episodes of community-acquired LRTI were identified in 448,469 patients (median number of episodes=1, IQR:1-2). The median age at diagnosis was 76 (IQR:70-82) years. Crude overall LRTI incidence was 122.93 episodes/1000 person-years (IQR:122.49-123.37/1000 person-years); incidence generally increased over time with some fluctuations, from a low of 100.96 (1997) to a peak of 148.04/1000 person-years (2008), and was similar in men and women (Figure 1a & Table S1). After standardising for age the increase over time was less marked, and a higher rate in men than women was revealed (Figure 1a & Table S2).

**Figure 2:**
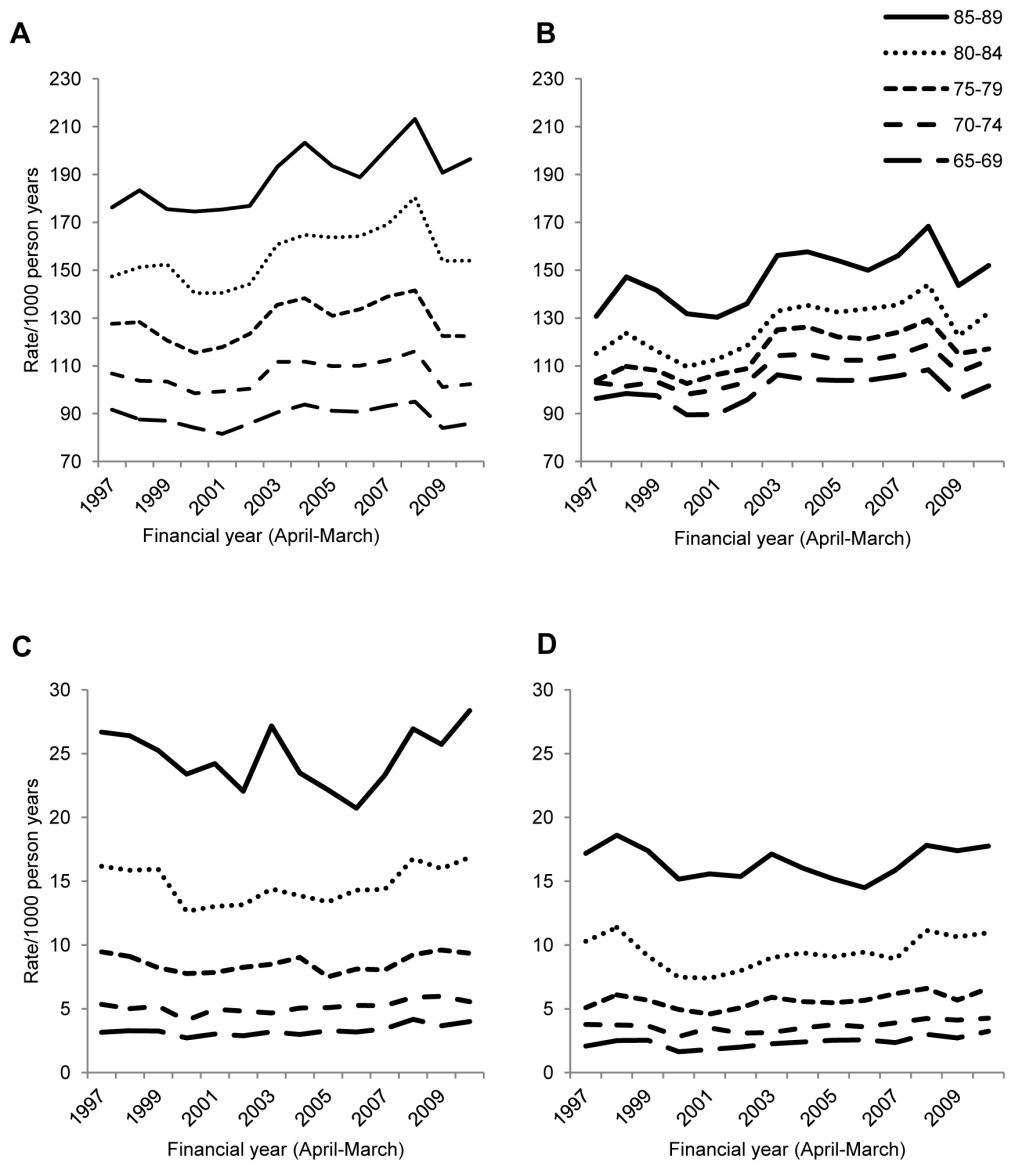
Incidence of LRTI and CAP by age and sex over time. Incidence by age of LRTI in a) men, b) women and CAP in c) men, d) women over time.

The difference between the sexes was also apparent in the age-stratified rates (Figure 2a,b), particularly in older age groups. Incidence increased with age, doubling between the 65-69 and 85-89 age groups, and fluctuations over time were more marked at older ages. Age-stratified results were not presented graphically for those aged 90+, as the age structure of this group varied over time, making the results difficult to interpret (Table S1).

Inclusion of COPD exacerbation codes did not change the overall pattern of LRTI incidence over time, but increased the rates in both sexes by around 7% (Table S3 & Figure S1).

A total of 64,978 CAP episodes were identified in 58,772 patients. CAP patients were generally older than those with LRTI (median age=81 years, IQR:75-87). Overall incidence of CAP was 7.99/1000 person-years (IQR:7.92-8.07/1000 person-years), was somewhat higher in men than women and increased slightly over time (Figure 1b, Table S4). After standardising for age, the increase was no longer apparent and the higher rate in men than women was accentuated (Figure 1b & Table S2). CAP rates increased with age with the rate in the 85-89 years group over seven times that of the 65-69 year olds (Figure 2c,d). Women’s CAP incidence was comparable to that of men aged five years younger.

### Incidence by region and deprivation

**Figure 3:**
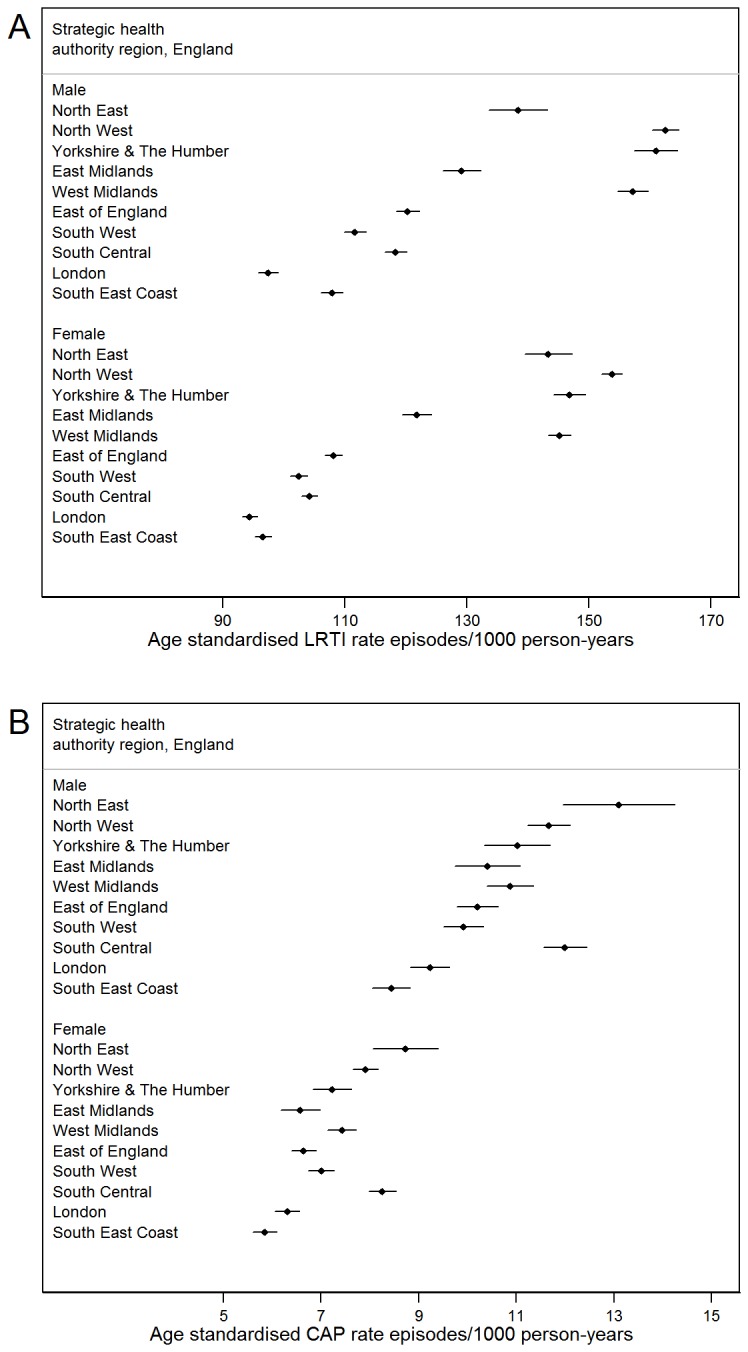
Age-standardised incidence of LRTI and CAP by region and sex. Age-standardised incidence of a) LRTI and b) CAP by region and sex. Standardised to UK population, mid-year 2004.

Incidence of both LRTI and CAP varied markedly by English region (Figure 3). Age-standardised incidence of LRTI was higher among the North and Midland regions than the South, and was highest in the North West. For CAP, high rates were seen in the North East, but also in the South Central region. For both conditions, the lowest rates were in London and the South East Coast. Rates are only presented for England; rates for Scotland, Northern Ireland and Wales were not comparable, due to lack of linked HES data outside England.

**Figure 4:**
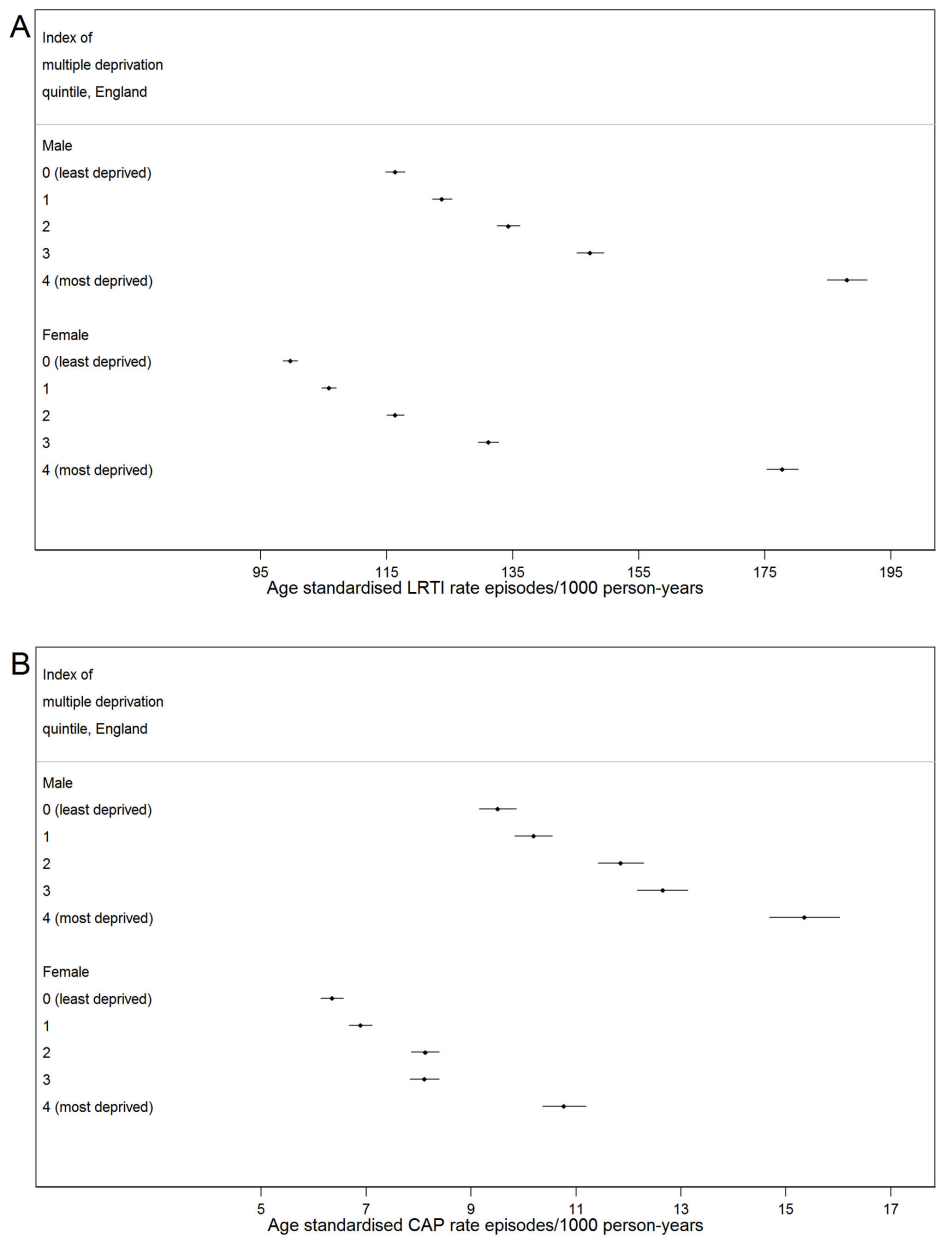
Age-standardised incidence of LRTI and CAP by IMD quintile and sex. Age-standardised incidence of a) LRTI and b) CAP by index of multiple deprivation quintile and sex. Standardised to UK population, mid-year 2004.

Incidence of both LRTI and CAP increased with increasing deprivation, with a marked difference between IMD quintiles three and four (the most deprived, Figure 4). This pattern was present for both men and women, and remained after standardising for age.

### Antibiotic treatment of LRTI and CAP

More than three-quarters of LRTI patients were prescribed antibiotics by their GP on the day their illness-episode was diagnosed; 7.8% did not receive antibiotics but were hospitalised (Table 2). Over half of CAP patients were hospitalised on the day of diagnosis without a GP-prescribed antibiotic on that day (58.2%), with death on the index date without antibiotics or hospitalisation (13.0%) more common than antibiotic receipt (9.9%). A larger percentage of CAP (12.7%) than LRTI episodes (11.0%) had no antibiotic or hospitalisation records in the 29 days after the index date (Table 2). Of these CAP episodes, only 10% had received antibiotics in the previous week. Penicillins, macrolides and cephalosporins were the most commonly prescribed antibiotics on the day for both conditions (Table 3).

**Table 2:** Timing of antibiotic prescriptions and other outcomes around the index date of LRTI and CAP illness-episodes.

		**LRTI**		**CAP**	
**Timing of treatment**	**Treatment received (all mutually exclusive)**		Antibiotics prescribed in 7 days before index date		Antibiotics prescribed in 7 days before index date
		**Total (%)**	**n (%)**	**Total (%)**	**n (%)**
**On index date**	Antibiotics prescribed	738794 (75.9)	22541 (3.1)	6412 (9.9)	777 (12.1)
	Patient hospitalised (no GP antibiotics)	75453 (7.8)	8013 (10.6)	37830 (58.2)	4980 (13.2)
	Patient died (no GP antibiotics/hospitalisation)	8094 (0.8)	937 (11.6)	8476 (13.0)	1450 (17.1)
**1-7 days after index date**	Antibiotics prescribed	16116 (1.7)	1776 (11.0)	788 (1.2)	157 (19.9)
	Patient hospitalised (no GP antibiotics)	5461 (0.6)	671 (12.3)	961 (1.5)	168 (17.5)
	Patient died (no GP antibiotics/hospitalisation)	722 (<0.1)	123 (17.0)	399 (0.6)	102 (25.6)
**8-28 days after index date**	Antibiotics prescribed	17406 (1.8)	2198 (12.6)	1155 (1.8)	208 (18.0)
	Patient hospitalised (no GP antibiotics)	4497 (0.5)	27 (9.3)	610 (0.9)	72 (11.8)
	Patient died (no GP antibiotics/hospitalisation)	291 (<0.1)	423 (9.1)	89 (0.1)	21 (23.6)
**No treatment recorded on index date or subsequent 28 days**		107287 (11.0)	7355 (6.9)	8258 (12.7)	855 (10.4)
**Total**		**974121 (100.0)**	**44064 (4.5)**	**64978 (100.0)**	**8790 (13.5)**

**Table 3:** Variety of antibiotic (by BNF sub-chapter) prescribed on the LRTI/CAP index date.

**Antibiotic variety**	**Number of varieties prescribed**	
**(by BNF^a^ sub-chapter)**		
	**LRTI**	**CAP**
	n (%)	n (%)
**Penicillins**	511253 (69.2)	3662 (57.1)
**Cephalosporins**	64609 (8.8)	610 (9.5)
**Tetracyclines**	36785 (5.0)	218 (3.4)
**Aminoglycosides**	18 (<0.1)	1 (<0.1)
**Macrolides**	125343 (17.0)	1665 (26.0)
**Clindamycin**	22 (<0.1)	3 (<0.1)
**Others**	130 (<0.1)	4 (<0.1)
**Sulphonamides**	10577 (1.4)	80 (1.3)
**Metronidazole**	542 (<0.1)	16 (0.3)
**Quinolones**	28812 (3.9)	522 (8.1)
**Nitrofurantoin & Methenamine**	692 (0.1)	21 (0.3)
**Two or more antibiotics**	25 (<0.1)	0 (0.0)
**Total varieties of Antibiotic**	778808 (105.4*)	6802 (106.0*)
**Patients prescribed to**	738794	6412

^a^ British National Formulary

^*^ Total is more than 100% as some patients were prescribed more than one variety of antibiotic

## Discussion

This is the first study to provide detailed estimates of the burden of community-acquired LRTI and CAP among older UK individuals over a prolonged time period. We have shown that the incidence of LRTI and CAP increases markedly with age within this older population. Those aged 85-89 years had double the rate of LRTI and seven times more CAP illness-episodes than those aged 65-69 years, and rates were predominantly higher in men than women of the same age group. Incidence varied between regions of England, with rates in the North generally higher than the South. We also found striking differences by IMD quintile, with incidence in the most deprived quintile around 70% higher than the lowest quintile for both LRTI and CAP. There was an increase in incidence of both diseases over the study period, although rates did fluctuate somewhat. The increase was attenuated after age-standardisation, indicating that the rise was largely due to population aging. LRTIs were most commonly treated by an antibiotic prescription from a GP, whereas over half of CAP patients did not receive a GP antibiotic prescription but were hospitalised on the illness index date.

Up to now there has been relatively little detailed information on the incidence of these infections among the UK’s older population. Importantly, our exclusion of person-time not at risk, and identification of potentially hospital-acquired illness provides more accurate estimates of community-acquired infection than previously presented for UK older adults [1,7]. The derivation of illness-episodes allowed us to combine repeat consultations for one illness, giving a better measure of new illness compared to studies which included all consultations or restricted analyses to the first consultation in a year [7,8]. The incidence of LRTI we present is up to double that reported in previous UK studies [1,8]. A key factor in the age-adjusted increase in incidence in our study is likely to be the improved survival of patients with co-morbidities, resulting in a higher prevalence of patients at increased risk of infection over time. Our CAP incidence rates are almost 20% higher than previously reported in a similar UK GP population during a slightly earlier period, although with similar trends by sex and IMD [7].

Comparison of our findings with those from elsewhere in Europe are restricted by the paucity of large European studies of either LRTI or CAP set specifically in an older population, by methodological differences and limitations of some studies, and by real variation in the underlying risk profile of the populations studied. Among LRTI studies, incidence of first LRTI among 85-90 year olds in a municipality in the Netherlands during the first half of our study period estimated a considerably lower rate (93.8/1000 person years) than that we present [20]. This small study only included patients’ first episode of LRTI in incidence estimates and did not exclude person-time spent in hospital, which may explain much of the difference. Estimates from the Second Dutch National Survey of General Practice (2000-2002) were also lower than ours (70/1000 person-years among those ≥75); again, person-time not at risk was not excluded, and a different coding system for LRTI was used [21].

Rates of CAP among older adults in Europe in the last 30 years have varied widely, both between and within countries. Some of these were small regional studies, and/or were restricted to either hospital or primary care settings. For example, a Spanish cohort study (2002-2005) of individuals aged ≥65 years set in the Tarragona region which did include both outpatient and hospitalised cases estimated CAP incidence at 14/1000 person years, twice that reported in this paper; higher incidence was largely among 65-74 year olds [6]. An active surveillance program for CAP was established before the start of the Spanish study, with primary care physicians encouraged to register all CAP cases confirmed radiographically. This could have changed primary care physicians’ diagnostic practices for CAP. In addition, a high proportion of individuals sought care directly from hospital and not from their general practitioner. This may have resulted in a somewhat more frequent categorisation of LRTI cases as CAP compared to our study population, in which some younger less severe cases would have been diagnosed and treated in primary care (where radiological investigations for suspected CAP are uncommon). In contrast, our CAP rates are considerably higher than those presented by another small Spanish study set in the Barcelona region in a slightly earlier study period, which estimated CAP incidence as 3.16/1000 among those aged ≥65 [22]. Neither Spanish study removed person-time not at risk of CAP nor age-standardised their overall rates. A large Italian study gave a lower CAP incidence estimate of 4.8/1000 population among those aged ≥65 years, but was restricted to hospitalised cases [23]. Interestingly, a large German study that also included only hospitalised CAP patients reported CAP incidence in those aged ≥60 of 7.65/1000 population, similar to our findings [24]. As with our study, the German and Italian studies reported consistently higher rates in men and sharply rising incidence with increasing age. Incidence of CAP was higher still in a small Finnish study of both hospitalised and non-hospitalised CAP in 1981/2 at 19.9/1000 population [25]. The difference between this finding and ours may be due in part to an earlier study period and climatic differences. Comparisons of our CAP incidence findings with those from the USA are less meaningful, as in the US patients in long-term residential care with pneumonia are not included with CAP but classified separately as having healthcare-associated pneumonia, which is not recommended practice in Europe [26].

During our study period routine vaccination for all older adults against seasonal influenza and pneumococcal disease was introduced, in 2000 and 2003 respectively. Yearly uptake of influenza vaccine has increased from 65% in 2000 and has remained between 71% and 75% since 2003 [27,28]. Coverage of pneumococcal vaccination (PPV23) has increased steadily from 29% of ≥65 year olds in 2003 to an estimated 70.5% by 31 March 2011 [29,30]. Thus our findings show rising LRTI and CAP incidence despite increasing levels of influenza and pneumococcal vaccine coverage. This might be in part because effectiveness of the PPV23 vaccine among older individuals appears to be limited [31].

As lower respiratory tract infections are caused by a number of pathogens whose circulation and severity of resulting disease varies from year to year, it is no surprise that LRTI incidence fluctuates somewhat over time. However, the peak LRTI incidence shown here in 2008 has not been reported elsewhere. National reported laboratory data for England and Wales do not show peaks in isolates of *Mycoplasma pneumoniae*, respiratory syncitial virus or influenza A or B in 2008 [32,33]. It should be noted that this peak is emphasised by the decrease in 2009, which could have resulted from low susceptibility among older individuals to the pandemic strain of influenza circulating in the 2009/10 season [34].

A high proportion of individuals with LRTI were given an antibiotic prescription on the index date, similar to a previous report from 1995–2000 [13]. It has been shown that among patients aged ≥65 presenting with an LRTI, 4% of those not prescribed antibiotics on the index date were diagnosed with pneumonia in the next month, compared to 1.5% of those prescribed antibiotics [35]. Higher antibiotic prescribing rates in older people may be to prevent worsening of LRTI or deterioration of co-morbidities, in line with recently issued guidance [36].

The increase in LRTI and CAP with age is unsurprising given age-related immunosenescence, and the growing prevalence of co-morbidities within older groups. The previously unreported regional differences between the North and South of England shown in this study may be due to interrelated socioeconomic and other factors, such as smoking and nutritional habits, as well as extent of co-morbidities and lower winter temperatures. It is notable that the regions with the highest age-standardised LRTI rates (North West, Yorkshire and the Humber, West Midlands and females in the North East) also have high reported prevalence of smoking [37]. The high level of CAP (but not LRTI) found in the South Central region was unexpected, and we cannot currently explain this; it does not appear to be due to a higher proportion of ‘oldest old’. We cannot exclude the possibility that some of the regional variation in CAP is due to different diagnostic preferences geographically in categorising LRTI as CAP. However, we think it unlikely that this would explain all of the marked variation seen.

Hospitalisation without a GP antibiotic prescription was the primary intervention for 58% of CAP episodes in this study. Previous reports estimated that a third of all pneumonia patients are treated in hospital [38]; we would expect this to be higher among our older population. Penicillins and macrolides were the most prescribed antibiotics, in line with British Thoracic Society guidelines issued at the time [39]. CAP cases who died on the index date (13%) will have included some death notifications received by the GP. However, 12.7% of CAP cases had no record of treatment or death on the index date or the following four weeks, and only 10% of these patients had received antibiotics in the preceding week. Reasons for this may include high-risk patients taking previously prescribed prophylactic antibiotics, or prescription of antibiotics during a home visit that were incompletely captured in the electronic record.

Our study has many strengths, being a large, population-based study of over 1.4 million patients’ primary care records, with additional information on hospital admissions for 59% of patients. The addition of linked HES admission/discharge dates allowed better differentiation between potentially hospital- and community-acquired illness-episodes. It also enabled exclusion of person-time not at risk from the incidence calculation. Older adults spend more time in hospital than their younger counterparts, making this an important consideration. HES linkage was not available for the whole study population, and so we could not remove person-time at risk from the denominator of all patients. This may have led to a slight underestimation of incidence. However, we did use hospitalisation codes recorded in CPRD to exclude potentially hospital-acquired infections from the CPRD-only subset of the data.

In primary care settings in the UK, GPs often diagnose pneumonia without an x-ray, which may have led to some CAP cases being categorised as LRTI and vice-versa. Misclassification between other conditions (e.g. chronic respiratory disease) and LRTI may have occurred in a minority of patients over time, but is not likely to have favoured one condition over the other. Clinical guidelines for the diagnosis of LRTI did not change substantially during the study period but we cannot exclude the possibility that increased awareness and variation in clinical practice could have contributed in part to some of the upward trend observed. Thus our estimates reflect those of GP clinical opinion, in line with previous studies [1,7,35,40].

We used an episode structure for illnesses due to the high consultation rate among our study population [41]. The 28-day period free from LRTI consultations specified as necessary before a new episode could begin was chosen to be similar to previous UK studies, which excluded patients if they had an LRTI diagnosis up to 28 days before the index date [8,40]. It is possible that using this period excluded a few new illnesses from the numerator of our rate, and also excluded some person-time at risk. The 14-day exclusion period we placed after any hospitalisation is commonly used [14,16–19], but again may have excluded some new community-acquired episodes.

## Conclusions

Community-acquired LRTI and CAP are important causes of morbidity and mortality in the aging UK population. Our new estimates show that the summary incidence of LRTI and CAP commonly presented for the ≥65 age group considerably underestimates UK disease rates in the higher ages within this group. It is important that variations in LRTI and CAP incidence in older individuals by age, region and IMD are taken into account in future health planning in the UK. Routine data such as these are used in many countries to assess disease burden. Given our findings, our methodology is likely to be highly relevant to other countries with aging populations, so that they can obtain more accurate incidence estimates of these important infections.

## Supporting Information

Table S1 **Community-acquired LRTI incidence rates overall and over time by sex, age, region and IMD quintile.**
(DOC)

Table S2 **Age-standardised incidence of LRTI and CAP by year, region and IMD quintile.** Standardised to UK population, mid-year 2004.
(DOC)

Table S3 **Community-acquired LRTI incidence including COPD exacerbations over time by sex, age, region and IMD quintile.**
(DOC)

Figure S1 **Age standardised incidence of LRTI including COPD exacerbation codes by sex over time.** Standardised to UK population, mid-year 2004.
(DOCX)

Table S4 **Community-acquired pneumonia incidence rates overall and over time by sex, age, region and IMD quintile.**
(DOC)
