# Supplementary material for: Incidence of Community-Acquired Lower Respiratory Tract Infections and Pneumonia among Older Adults in the United Kingdom: A Population-Based Study
Source: PLoS One. 2013 Sep 11;8(9):e75131. doi: 10.1371/journal.pone.0075131 (PMC3770598; doi:10.1371/journal.pone.0075131)
Supplement: Table S1 — Community-acquired LRTI incidence rates overall and over time by sex, age, region and IMD quintile. (DOC) [file pone.0075131.s001.doc]

**Table S1. Community-acquired LRTI incidence rates overall and over time by sex, age, region and IMD quintile.**

|  |  | **Sex** | | **Age (years)** | | | | | |
| --- | --- | --- | --- | --- | --- | --- | --- | --- | --- |
|  |  | Male | Female | 65-69 | 70-74 | 75-79 | 80-84 | 85-89 | ≥90 |
| **Overall** | 122.93 | 121.76 | 123.83 | 92.21 | 107.40 | 126.07 | 151.37 | 187.91 | 262.87 |
| **95% CI** | 122.49-123.37 | 121.1-122.42 | 123.24-124.42 | 91.7-92.71 | 106.8-108 | 125.33-126.81 | 150.36-152.39 | 186.32-189.49 | 259.79-265.94 |
| **1997** | 100.96 | 100.87 | 100.89 | 94.10 | 104.72 | 113.58 | 126.88 | 144.38 | 170.43 |
|  | 99.91-102.01 | 99.27-102.48 | 99.51-102.28 | 92.01-96.19 | 102.38-107.06 | 110.91-116.26 | 123.37-130.38 | 139.49-149.27 | 163.18-177.69 |
| **1998** | 104.37 | 101.35 | 106.54 | 93.24 | 102.54 | 117.40 | 133.64 | 158.26 | 177.00 |
|  | 103.35-105.38 | 99.82-102.88 | 105.18-107.9 | 91.29-95.19 | 100.37-104.72 | 114.88-119.92 | 130.16-137.13 | 153.4-163.12 | 170.03-183.97 |
| **1999** | 105.40 | 102.74 | 107.32 | 92.49 | 103.44 | 113.37 | 129.50 | 152.10 | 186.11 |
|  | 104.44-106.36 | 101.3-104.19 | 106.04-108.61 | 90.7-94.29 | 101.42-105.46 | 111.1-115.65 | 126.32-132.69 | 147.72-156.47 | 179.44-192.79 |
| **2000** | 102.13 | 100.69 | 103.14 | 86.91 | 98.31 | 108.03 | 121.03 | 144.89 | 173.51 |
|  | 101.22-103.03 | 99.32-102.05 | 101.94-104.34 | 85.29-88.54 | 96.46-100.15 | 105.92-110.13 | 118.23-123.82 | 140.87-148.92 | 167.46-179.56 |
| **2001** | 105.90 | 104.07 | 107.23 | 85.74 | 99.73 | 111.24 | 123.05 | 144.29 | 183.92 |
|  | 105-106.81 | 102.7-105.44 | 106.03-108.44 | 84.18-87.3 | 97.92-101.54 | 109.14-113.34 | 120.37-125.72 | 140.38-148.2 | 177.86-189.98 |
| **2002** | 112.22 | 110.64 | 113.39 | 91.00 | 101.98 | 115.03 | 128.12 | 148.88 | 185.77 |
|  | 111.3-113.15 | 109.23-112.04 | 112.16-114.63 | 89.42-92.57 | 100.2-103.77 | 112.92-117.14 | 125.49-130.75 | 144.9-152.85 | 179.79-191.74 |
| **2003** | 127.30 | 124.11 | 129.72 | 98.61 | 113.08 | 129.55 | 143.43 | 167.98 | 211.54 |
|  | 126.3-128.3 | 122.61-125.61 | 128.38-131.05 | 96.99-100.23 | 111.19-114.97 | 127.29-131.8 | 140.69-146.18 | 163.65-172.32 | 205.14-217.94 |
| **2004** | 131.97 | 130.66 | 133.00 | 99.20 | 113.45 | 131.45 | 146.52 | 172.24 | 223.38 |
|  | 130.95-132.99 | 129.11-132.21 | 131.64-134.35 | 97.6-100.8 | 111.57-115.32 | 129.2-133.7 | 143.78-149.26 | 167.88-176.6 | 216.8-229.96 |
| **2005** | 131.76 | 131.06 | 132.34 | 97.69 | 111.24 | 125.93 | 144.46 | 166.92 | 222.45 |
|  | 130.74-132.78 | 129.51-132.62 | 130.99-133.69 | 96.12-99.26 | 109.4-113.08 | 123.76-128.1 | 141.74-147.19 | 162.84-171 | 215.89-229 |
| **2006** | 134.09 | 134.70 | 133.72 | 97.57 | 111.26 | 126.59 | 145.70 | 162.82 | 223.53 |
|  | 133.05-135.12 | 133.11-136.3 | 132.35-135.08 | 96-99.14 | 109.43-113.1 | 124.41-128.77 | 142.94-148.46 | 158.93-166.7 | 216.92-230.14 |
| **2007** | 139.88 | 142.07 | 138.36 | 99.66 | 113.43 | 130.54 | 148.68 | 171.04 | 234.79 |
|  | 138.81-140.95 | 140.42-143.73 | 136.96-139.77 | 98.08-101.25 | 111.58-115.28 | 128.32-132.76 | 145.86-151.49 | 167.11-174.97 | 227.84-241.73 |
| **2008** | 148.04 | 149.65 | 147.02 | 101.86 | 117.61 | 134.62 | 158.30 | 183.45 | 267.05 |
|  | 146.93-149.16 | 147.92-151.37 | 145.56-148.49 | 100.26-103.47 | 115.71-119.51 | 132.33-136.91 | 155.34-161.26 | 179.34-187.55 | 259.28-274.82 |
| **2009** | 131.10 | 132.49 | 130.24 | 90.26 | 104.38 | 118.38 | 134.93 | 159.58 | 229.95 |
|  | 130.06-132.14 | 130.88-134.1 | 128.87-131.61 | 88.77-91.74 | 102.6-106.16 | 116.24-120.52 | 132.23-137.63 | 155.8-163.35 | 222.78-237.11 |
| **2010** | 137.30 | 135.46 | 138.90 | 93.95 | 107.69 | 119.46 | 140.93 | 167.30 | 240.21 |
|  | 136.21-138.38 | 133.81-137.1 | 137.46-140.35 | 92.42-95.48 | 105.85-109.53 | 117.27-121.64 | 138.13-143.74 | 163.33-171.27 | 233.03-247.38 |

|  | **Region** | | | | | | | | | |
| --- | --- | --- | --- | --- | --- | --- | --- | --- | --- | --- |
|  | North East | North West | Yorkshire & The Humber | East Midlands | West Midlands | East of England | South West | South Central | London | South East Coast |
| **Overall** | 145.47 | 158.81 | 156.37 | 126.43 | 154.29 | 115.72 | 110.27 | 114.11 | 96.84 | 104.30 |
| **95% CI** | 141.85-149.09 | 157.23-160.39 | 153.87-158.88 | 124.2-128.66 | 152.48-156.11 | 114.38-117.05 | 108.95-111.59 | 112.84-115.37 | 95.68-98 | 103.03-105.58 |
| **1997** | 104.47 | 134.85 | 110.13 | 93.82 | 111.37 | 91.68 | 94.63 | 96.80 | 90.03 | 78.03 |
|  | 97.94-111 | 131.47-138.23 | 105.65-114.61 | 89.45-98.18 | 107.77-114.96 | 88.69-94.68 | 91.15-98.1 | 93.12-100.47 | 86.8-93.26 | 74.85-81.21 |
| **1998** | 112.42 | 125.24 | 124.57 | 108.18 | 130.84 | 101.35 | 94.42 | 94.97 | 93.54 | 79.21 |
|  | 105.52-119.31 | 122.13-128.35 | 119.87-129.27 | 103.61-112.76 | 127.14-134.54 | 98.35-104.36 | 91.22-97.62 | 91.67-98.26 | 90.35-96.73 | 76.07-82.34 |
| **1999** | 135.29 | 125.99 | 125.62 | 98.18 | 146.35 | 97.61 | 98.25 | 98.01 | 85.66 | 84.53 |
|  | 127.49-143.09 | 122.97-129.01 | 120.96-130.28 | 94.05-102.31 | 142.49-150.21 | 94.8-100.41 | 95.28-101.22 | 95.04-100.98 | 82.8-88.51 | 81.52-87.53 |
| **2000** | 122.24 | 127.19 | 131.39 | 106.53 | 125.68 | 94.40 | 86.60 | 93.83 | 91.12 | 81.63 |
|  | 115.32-129.16 | 124.2-130.18 | 126.66-136.12 | 102.17-110.9 | 122.33-129.03 | 91.72-97.08 | 83.92-89.29 | 91.2-96.47 | 88.32-93.92 | 78.88-84.39 |
| **2001** | 136.47 | 126.64 | 150.14 | 112.37 | 132.35 | 104.34 | 97.19 | 103.33 | 96.77 | 90.76 |
|  | 128.95-143.99 | 123.73-129.55 | 145.08-155.19 | 107.93-116.8 | 128.89-135.81 | 101.52-107.15 | 94.31-100.08 | 100.63-106.04 | 93.95-99.59 | 87.89-93.64 |
| **2002** | 152.07 | 142.05 | 160.25 | 116.39 | 146.89 | 111.47 | 102.17 | 102.96 | 91.00 | 95.52 |
|  | 143.91-160.23 | 138.99-145.12 | 155.07-165.42 | 111.86-120.92 | 143.19-150.58 | 108.58-114.37 | 99.21-105.12 | 100.24-105.67 | 88.32-93.68 | 92.64-98.4 |
| **2003** | 148.22 | 165.30 | 182.43 | 134.71 | 165.18 | 121.73 | 115.81 | 115.33 | 98.48 | 104.90 |
|  | 140.12-156.33 | 161.91-168.69 | 176.7-188.16 | 129.73-139.69 | 161.21-169.14 | 118.66-124.8 | 112.64-118.99 | 112.43-118.23 | 95.65-101.31 | 101.9-107.9 |
| **2004** | 177.09 | 173.26 | 178.00 | 139.49 | 168.54 | 126.02 | 116.06 | 121.68 | 100.24 | 106.26 |
|  | 167.98-186.2 | 169.76-176.76 | 172.31-183.68 | 134.26-144.71 | 164.49-172.59 | 122.89-129.15 | 112.91-119.22 | 118.68-124.69 | 97.35-103.13 | 103.25-109.26 |
| **2005** | 159.09 | 174.18 | 173.41 | 144.65 | 167.85 | 133.99 | 116.01 | 120.20 | 97.75 | 107.35 |
|  | 150.54-167.64 | 170.66-177.7 | 167.79-179.02 | 139.26-150.05 | 163.76-171.93 | 130.71-137.26 | 112.87-119.16 | 117.2-123.2 | 94.92-100.59 | 104.34-110.37 |
| **2006** | 152.34 | 176.98 | 175.66 | 156.95 | 168.53 | 125.31 | 118.64 | 124.05 | 98.54 | 114.06 |
|  | 143.96-160.72 | 173.39-180.58 | 169.79-181.52 | 151.2-162.71 | 164.43-172.63 | 122.08-128.53 | 115.47-121.8 | 120.98-127.12 | 95.67-101.41 | 110.97-117.15 |
| **2007** | 162.59 | 186.16 | 180.05 | 154.15 | 170.63 | 129.19 | 126.68 | 126.05 | 106.05 | 117.67 |
|  | 154.12-171.06 | 182.4-189.91 | 173.95-186.16 | 148.31-160 | 166.43-174.83 | 125.79-132.58 | 123.38-129.99 | 123.03-129.07 | 103.09-109.01 | 114.53-120.8 |
| **2008** | 176.49 | 193.87 | 192.55 | 162.69 | 181.75 | 138.13 | 132.73 | 137.58 | 108.18 | 130.18 |
|  | 167.46-185.52 | 190-197.74 | 185.6-199.5 | 156.41-168.97 | 177.38-186.12 | 134.49-141.78 | 129.32-136.14 | 134.42-140.74 | 105.19-111.18 | 126.83-133.54 |
| **2009** | 162.08 | 177.40 | 170.31 | 135.91 | 162.97 | 123.46 | 113.54 | 116.89 | 96.91 | 117.51 |
|  | 153.38-170.79 | 173.71-181.09 | 163.04-177.57 | 129.98-141.85 | 158.89-167.06 | 119.99-126.94 | 110.44-116.64 | 114.04-119.74 | 94.13-99.7 | 114.4-120.62 |
| **2010** | 165.09 | 192.69 | 169.65 | 136.60 | 175.98 | 133.71 | 121.80 | 125.60 | 99.86 | 123.34 |
|  | 156.22-173.95 | 188.76-196.62 | 162.24-177.06 | 129.35-143.86 | 171.68-180.29 | 129.93-137.49 | 118.52-125.08 | 122.64-128.56 | 97.06-102.66 | 120.11-126.57 |

|  | **IMD Quintile** | | | | |
| --- | --- | --- | --- | --- | --- |
|  | 0 (least deprived) | 1 | 2 | 3 | 4 (most deprived) |
| **Overall** | 106.48 | 113.98 | 124.96 | 137.94 | 182.12 |
| **95% CI** | 105.39-107.57 | 112.86-115.1 | 123.63-126.28 | 136.4-139.47 | 179.79-184.46 |
| **1997** | 85.30 | 90.83 | 96.71 | 111.58 | 147.93 |
|  | 82.48-88.13 | 88.11-93.55 | 93.65-99.77 | 108.14-115.02 | 143.27-152.6 |
| **1998** | 92.70 | 97.59 | 103.73 | 113.91 | 141.04 |
|  | 89.93-95.47 | 94.88-100.3 | 100.71-106.75 | 110.58-117.25 | 136.68-145.4 |
| **1999** | 98.55 | 97.65 | 102.77 | 116.35 | 155.08 |
|  | 95.93-101.18 | 95.09-100.21 | 99.95-105.59 | 113.12-119.57 | 150.58-159.58 |
| **2000** | 89.87 | 94.66 | 101.23 | 114.68 | 150.27 |
|  | 87.52-92.23 | 92.29-97.03 | 98.56-103.9 | 111.6-117.77 | 145.97-154.56 |
| **2001** | 96.82 | 103.00 | 114.62 | 118.80 | 153.41 |
|  | 94.39-99.24 | 100.56-105.45 | 111.8-117.45 | 115.73-121.87 | 149.12-157.71 |
| **2002** | 97.81 | 107.35 | 116.83 | 129.44 | 175.75 |
|  | 95.39-100.22 | 104.86-109.83 | 113.97-119.69 | 126.23-132.65 | 171.08-180.42 |
| **2003** | 110.06 | 117.13 | 129.80 | 147.04 | 194.89 |
|  | 107.48-112.64 | 114.52-119.74 | 126.75-132.85 | 143.55-150.53 | 189.86-199.92 |
| **2004** | 111.16 | 122.91 | 136.51 | 149.93 | 201.40 |
|  | 108.58-113.74 | 120.23-125.6 | 133.35-139.68 | 146.37-153.49 | 196.21-206.6 |
| **2005** | 112.51 | 120.96 | 135.56 | 151.76 | 198.54 |
|  | 109.91-115.1 | 118.31-123.61 | 132.38-138.73 | 148.14-155.38 | 193.34-203.75 |
| **2006** | 114.92 | 125.28 | 139.07 | 151.50 | 195.91 |
|  | 112.29-117.54 | 122.58-127.98 | 135.85-142.29 | 147.87-155.14 | 190.7-201.12 |
| **2007** | 114.45 | 125.76 | 142.85 | 155.35 | 204.71 |
|  | 111.86-117.04 | 123.06-128.46 | 139.59-146.1 | 151.66-159.05 | 199.34-210.08 |
| **2008** | 125.74 | 135.11 | 151.18 | 167.15 | 217.22 |
|  | 123.01-128.46 | 132.3-137.93 | 147.8-154.56 | 163.26-171.04 | 211.58-222.85 |
| **2009** | 110.04 | 118.71 | 134.39 | 150.72 | 205.75 |
|  | 107.55-112.53 | 116.12-121.29 | 131.25-137.53 | 147.04-154.4 | 200.2-211.31 |
| **2010** | 114.76 | 126.32 | 140.00 | 155.29 | 219.00 |
|  | 112.17-117.36 | 123.61-129.03 | 136.72-143.27 | 151.47-159.1 | 213.2-224.81 |

IMD – index of multiple deprivation
