# Supplementary material for: Incidence of Community-Acquired Lower Respiratory Tract Infections and Pneumonia among Older Adults in the United Kingdom: A Population-Based Study
Source: PLoS One. 2013 Sep 11;8(9):e75131. doi: 10.1371/journal.pone.0075131 (PMC3770598; doi:10.1371/journal.pone.0075131)
Supplement: Table S2 — Age-standardised incidence of LRTI and CAP by year, region and IMD quintile. Standardised to UK population, mid-year 2004. (DOC) [file pone.0075131.s002.doc]

**Table S2. Age-standardised* incidence of LRTI and CAP by year, region and IMD** quintile.

|  | **LRTI Age-standardised* rate/1000 person years** | | | | | **CAP Age-standardised* rate/1000 person years** | | | | |
| --- | --- | --- | --- | --- | --- | --- | --- | --- | --- | --- |
|  | Men | | 95% CI | Women | 95% CI | Men | 95% CI | Women | | 95% CI |
| **Year** |  |  | |  |  |  |  |  |  | |
| **1997** | 122.40 | 120.31-124.5 | | 107.72 | 106.18-109.27 | 10.27 | 9.68-10.87 | 6.82 | 6.48-7.17 | |
| **1998** | 121.34 | 119.38-121.34 | | 112.11 | 110.62-113.6 | 9.91 | 9.36-10.46 | 7.51 | 7.17-7.85 | |
| **1999** | 120.24 | 118.44-122.05 | | 110.64 | 109.27-112.01 | 9.89 | 9.39-10.4 | 6.79 | 6.49-7.1 | |
| **2000** | 114.01 | 112.38-115.64 | | 103.87 | 102.63-105.11 | 8.31 | 7.88-8.74 | 5.56 | 5.3-5.81 | |
| **2001** | 115.00 | 113.42-116.59 | | 105.80 | 104.59-107.02 | 8.99 | 8.56-9.41 | 5.77 | 5.52-6.02 | |
| **2002** | 118.49 | 116.92-120.07 | | 110.16 | 108.94-111.37 | 8.78 | 8.37-9.19 | 5.83 | 5.58-6.08 | |
| **2003** | 129.80 | 128.16-131.44 | | 123.90 | 122.6-125.19 | 9.76 | 9.32-10.19 | 6.54 | 6.28-6.8 | |
| **2004** | 133.16 | 131.52-134.81 | | 124.75 | 123.47-126.03 | 9.47 | 9.05-9.89 | 6.62 | 6.36-6.88 | |
| **2005** | 129.94 | 128.34-131.55 | | 122.30 | 121.04-123.56 | 9.04 | 8.64-9.44 | 6.43 | 6.18-6.69 | |
| **2006** | 130.10 | 128.5-131.69 | | 122.13 | 120.87-123.39 | 9.36 | 8.96-9.76 | 6.43 | 6.17-6.68 | |
| **2007** | 134.88 | 133.25-136.51 | | 124.82 | 123.54-126.1 | 9.74 | 9.33-10.15 | 6.53 | 6.27-6.79 | |
| **2008** | 140.55 | 138.85-142.25 | | 131.36 | 130.04-132.69 | 11.09 | 10.65-11.53 | 7.65 | 7.37-7.93 | |
| **2009** | 122.79 | 121.22-124.36 | | 115.42 | 114.18-116.65 | 10.82 | 10.39-11.26 | 7.17 | 6.89-7.44 | |
| **2010** | 124.18 | 122.6-125.77 | | 121.24 | 119.95-122.52 | 11.00 | 10.57-11.44 | 7.64 | 7.36-7.92 | |
| **Region** |  |  | |  |  |  |  |  |  | |
| **North East** | 138.39 | 133.56-143.21 | | 143.34 | 139.49-147.2 | 13.10 | 11.95-14.24 | 8.73 | 8.05-9.4 | |
| **North West** | 162.52 | 160.3-164.74 | | 153.74 | 152.06-155.42 | 11.67 | 11.23-12.1 | 7.91 | 7.65-8.17 | |
| **Yorkshire & The Humber** | 160.95 | 157.44-164.47 | | 146.74 | 144.12-149.36 | 11.02 | 10.34-11.69 | 7.23 | 6.83-7.63 | |
| **East Midlands** | 129.12 | 126.01-132.23 | | 121.78 | 119.37-124.18 | 10.41 | 9.73-11.08 | 6.58 | 6.18-6.97 | |
| **West Midlands** | 157.17 | 154.67-159.68 | | 145.14 | 143.26-147.02 | 10.87 | 10.39-11.35 | 7.43 | 7.14-7.72 | |
| **East of England** | 120.29 | 118.41-122.17 | | 108.10 | 106.7-109.5 | 10.20 | 9.78-10.63 | 6.65 | 6.39-6.9 | |
| **South West** | 111.64 | 109.85-113.43 | | 102.41 | 101.02-103.81 | 9.92 | 9.5-10.33 | 7.01 | 6.74-7.27 | |
| **South Central** | 118.30 | 116.51-120.08 | | 104.20 | 102.89-105.52 | 12.00 | 11.55-12.45 | 8.25 | 7.97-8.53 | |
| **London** | 97.38 | 95.76-98.99 | | 94.41 | 93.11-95.71 | 9.23 | 8.82-9.63 | 6.31 | 6.06-6.56 | |
| **South East Coast** | 107.87 | 106.08-109.67 | | 96.57 | 95.22-97.91 | 8.44 | 8.05-8.83 | 5.85 | 5.61-6.09 | |
| **IMD Quintile** |  |  | |  |  |  |  |  |  | |
| **0 (least deprived)** | 116.31 | 114.75-117.86 | | 99.67 | 98.51-100.83 | 9.51 | 9.15-9.86 | 6.35 | 6.13-6.56 | |
| **1** | 123.75 | 122.17-125.33 | | 105.82 | 104.65-106.99 | 10.19 | 9.83-10.55 | 6.89 | 6.67-7.11 | |
| **2** | 134.28 | 132.43-136.13 | | 116.33 | 114.95-117.71 | 11.85 | 11.42-12.29 | 8.13 | 7.86-8.39 | |
| **3** | 147.27 | 145.13-149.41 | | 131.15 | 129.53-132.77 | 12.65 | 12.16-13.13 | 8.11 | 7.83-8.39 | |
| **4 (most deprived)** | 188.05 | 184.91-191.19 | | 177.79 | 175.26-180.31 | 15.35 | 14.68-16.02 | 10.77 | 10.36-11.19 | |

*Age-standardised-UK mid-year population estimates, 2004.

LRTI: lower respiratory tract infection

CAP: community-acquired pneumonia

IMD – index of multiple deprivation
