# Supplementary material for: Incidence of Community-Acquired Lower Respiratory Tract Infections and Pneumonia among Older Adults in the United Kingdom: A Population-Based Study
Source: PLoS One. 2013 Sep 11;8(9):e75131. doi: 10.1371/journal.pone.0075131 (PMC3770598; doi:10.1371/journal.pone.0075131)
Supplement: Table S3 — Community-acquired LRTI incidence including COPD exacerbations over time by sex, age, region and IMD quintile. (DOC) [file pone.0075131.s003.doc]

**Table S3. Community-acquired LRTI incidence including COPD exacerbations over time by sex, age, region and IMD quintile.**

|  |  | **Sex** | | **Age (years)** | | | | | |
| --- | --- | --- | --- | --- | --- | --- | --- | --- | --- |
|  | Overall | Male | Female | 65-69 | 70-74 | 75-79 | 80-84 | 85-89 | ≥90 |
| **Overall** | 132.06 | 132.25 | 131.90 | 96.98 | 115.93 | 138.56 | 167.02 | 205.94 | 283.39 |
| **95% CI** | 131.57-132.54 | 131.52-132.99 | 131.25-132.54 | 96.45-97.51 | 115.29-116.57 | 137.75-139.37 | 165.9-168.14 | 204.19-207.69 | 280.01-286.77 |
| **1997** | 102.99 | 103.13 | 102.67 | 97.44 | 108.89 | 117.79 | 131.31 | 147.66 | 172.24 |
|  | 101.93-104.05 | 101.51-104.75 | 101.27-104.07 | 95.29-99.6 | 106.47-111.31 | 115.03-120.55 | 127.7-134.92 | 142.68-152.64 | 164.92-179.55 |
| **1998** | 107.20 | 104.37 | 109.17 | 96.76 | 108.03 | 122.73 | 138.61 | 162.71 | 178.74 |
|  | 106.17-108.24 | 102.82-105.92 | 107.79-110.55 | 94.75-98.77 | 105.77-110.3 | 120.12-125.35 | 135.02-142.2 | 157.73-167.68 | 171.71-185.77 |
| **1999** | 108.66 | 106.25 | 110.33 | 96.58 | 108.78 | 118.80 | 134.70 | 156.84 | 187.88 |
|  | 107.68-109.64 | 104.77-107.72 | 109.02-111.64 | 94.72-98.44 | 106.67-110.88 | 116.44-121.16 | 131.41-137.98 | 152.35-161.32 | 181.16-194.61 |
| **2000** | 106.13 | 105.18 | 106.72 | 90.83 | 104.15 | 114.84 | 126.44 | 149.35 | 176.59 |
|  | 105.2-107.05 | 103.78-106.59 | 105.48-107.95 | 89.15-92.52 | 102.22-106.08 | 112.63-117.05 | 123.54-129.33 | 145.22-153.48 | 170.45-182.72 |
| **2001** | 110.88 | 109.64 | 111.72 | 90.17 | 106.02 | 118.29 | 130.46 | 149.88 | 187.60 |
|  | 109.94-111.82 | 108.22-111.06 | 110.48-112.96 | 88.54-91.79 | 104.12-107.91 | 116.09-120.49 | 127.66-133.25 | 145.85-153.91 | 181.45-193.74 |
| **2002** | 118.37 | 117.62 | 118.87 | 96.00 | 109.20 | 123.67 | 135.77 | 154.99 | 188.69 |
|  | 117.41-119.33 | 116.15-119.09 | 117.59-120.14 | 94.35-97.64 | 107.32-111.09 | 121.43-125.9 | 133.01-138.52 | 150.88-159.1 | 182.64-194.74 |
| **2003** | 135.09 | 133.35 | 136.38 | 104.36 | 121.73 | 139.58 | 152.51 | 175.13 | 216.31 |
|  | 134.04-136.13 | 131.77-134.94 | 134.99-137.77 | 102.67-106.06 | 119.73-123.73 | 137.19-141.97 | 149.62-155.4 | 170.65-179.61 | 209.79-222.82 |
| **2004** | 141.46 | 141.89 | 141.16 | 105.96 | 122.82 | 143.08 | 157.15 | 180.19 | 229.10 |
|  | 140.38-142.54 | 140.23-143.54 | 139.75-142.58 | 104.28-107.65 | 120.83-124.81 | 140.68-145.49 | 154.25-160.04 | 175.67-184.71 | 222.39-235.81 |
| **2005** | 142.54 | 143.99 | 141.54 | 104.71 | 121.01 | 138.39 | 156.13 | 176.86 | 228.82 |
|  | 141.46-143.63 | 142.31-145.67 | 140.11-142.96 | 103.05-106.37 | 119.05-122.97 | 136.05-140.72 | 153.24-159.02 | 172.59-181.12 | 222.12-235.52 |
| **2006** | 146.43 | 149.37 | 144.35 | 105.11 | 122.53 | 139.74 | 157.93 | 173.59 | 230.53 |
|  | 145.31-147.54 | 147.64-151.11 | 142.9-145.81 | 103.45-106.78 | 120.56-124.51 | 137.39-142.09 | 155-160.87 | 169.5-177.67 | 223.75-237.31 |
| **2007** | 153.88 | 159.00 | 150.26 | 107.82 | 125.19 | 145.17 | 162.54 | 182.49 | 243.09 |
|  | 152.72-155.04 | 157.18-160.81 | 148.77-151.76 | 106.13-109.5 | 123.19-127.19 | 142.76-147.58 | 159.52-165.55 | 178.35-186.63 | 235.95-250.24 |
| **2008** | 164.29 | 169.30 | 160.84 | 111.09 | 131.21 | 150.71 | 174.46 | 195.47 | 275.88 |
|  | 163.08-165.51 | 167.38-171.21 | 159.26-162.42 | 109.37-112.81 | 129.14-133.29 | 148.2-153.21 | 171.26-177.65 | 191.14-199.79 | 267.89-283.87 |
| **2009** | 147.23 | 151.77 | 144.13 | 99.12 | 117.72 | 134.10 | 150.20 | 172.31 | 238.24 |
|  | 146.09-148.38 | 149.97-153.57 | 142.64-145.62 | 97.52-100.72 | 115.77-119.68 | 131.74-136.46 | 147.26-153.14 | 168.3-176.32 | 230.87-245.62 |
| **2010** | 156.15 | 157.56 | 155.42 | 104.10 | 123.21 | 138.42 | 158.71 | 180.61 | 248.54 |
|  | 154.95-157.36 | 155.7-159.43 | 153.84-157 | 102.44-105.76 | 121.17-125.25 | 135.97-140.87 | 155.63-161.79 | 176.39-184.84 | 241.15-255.93 |

|  | **Region** | | | | | | | | | |
| --- | --- | --- | --- | --- | --- | --- | --- | --- | --- | --- |
|  | North East | North West | Yorkshire & The Humber | East Midlands | West Midlands | East of England | South West | South Central | London | South East Coast |
| **Overall** | 162.65 | 174.90 | 167.21 | 137.18 | 166.00 | 123.46 | 117.59 | 121.05 | 102.81 | 110.23 |
| **95% CI** | 158.54-166.76 | 173.13-176.68 | 164.47-169.94 | 134.72-139.65 | 164.01-168 | 122.01-124.91 | 116.15-119.02 | 119.68-122.42 | 101.56-104.06 | 108.86-111.6 |
| **1997** | 109.65 | 140.62 | 112.76 | 96.63 | 113.58 | 92.99 | 95.77 | 98.12 | 91.64 | 79.07 |
|  | 102.93-116.38 | 137.14-144.09 | 108.22-117.3 | 92.18-101.07 | 109.95-117.22 | 89.97-96.01 | 92.28-99.25 | 94.42-101.82 | 88.38-94.9 | 75.86-82.28 |
| **1998** | 118.58 | 133.60 | 128.21 | 111.55 | 133.46 | 104.20 | 96.52 | 96.31 | 95.02 | 80.62 |
|  | 111.46-125.71 | 130.36-136.85 | 123.42-133 | 106.88-116.23 | 129.72-137.21 | 101.14-107.26 | 93.28-99.76 | 92.99-99.63 | 91.8-98.25 | 77.45-83.79 |
| **1999** | 143.51 | 135.32 | 129.80 | 100.77 | 149.58 | 100.68 | 100.70 | 99.92 | 87.92 | 86.44 |
|  | 135.39-151.62 | 132.15-138.49 | 125.04-134.57 | 96.56-104.97 | 145.66-153.49 | 97.82-103.54 | 97.69-103.72 | 96.91-102.92 | 85.02-90.82 | 83.39-89.49 |
| **2000** | 130.13 | 135.65 | 138.19 | 111.04 | 131.86 | 97.90 | 90.03 | 95.86 | 93.92 | 83.63 |
|  | 122.9-137.36 | 132.52-138.78 | 133.29-143.08 | 106.54-115.54 | 128.4-135.33 | 95.16-100.65 | 87.28-92.78 | 93.18-98.53 | 91.06-96.78 | 80.83-86.43 |
| **2001** | 149.33 | 136.90 | 156.42 | 118.35 | 139.05 | 108.12 | 100.71 | 106.57 | 100.91 | 92.94 |
|  | 141.3-157.37 | 133.83-139.98 | 151.21-161.64 | 113.73-122.97 | 135.46-142.63 | 105.23-111.01 | 97.75-103.67 | 103.8-109.34 | 98-103.82 | 90.01-95.86 |
| **2002** | 167.10 | 155.44 | 168.93 | 123.41 | 155.76 | 115.93 | 106.64 | 106.84 | 95.61 | 98.32 |
|  | 158.32-175.89 | 152.15-158.72 | 163.53-174.32 | 118.67-128.16 | 151.89-159.62 | 112.94-118.93 | 103.59-109.69 | 104.05-109.64 | 92.84-98.39 | 95.37-101.27 |
| **2003** | 165.73 | 181.23 | 192.46 | 145.16 | 175.62 | 128.86 | 121.47 | 120.09 | 104.48 | 108.93 |
|  | 156.9-174.56 | 177.58-184.89 | 186.47-198.46 | 139.87-150.46 | 171.46-179.78 | 125.65-132.07 | 118.18-124.76 | 117.09-123.08 | 101.52-107.43 | 105.84-112.01 |
| **2004** | 195.80 | 190.95 | 191.22 | 151.80 | 182.43 | 135.40 | 122.06 | 128.23 | 106.87 | 111.97 |
|  | 185.9-205.71 | 187.16-194.74 | 185.2-197.24 | 146.2-157.4 | 178.12-186.74 | 132.08-138.71 | 118.78-125.34 | 125.09-131.36 | 103.84-109.9 | 108.84-115.1 |
| **2005** | 175.79 | 192.56 | 189.46 | 160.46 | 183.45 | 144.96 | 122.55 | 128.88 | 105.34 | 113.05 |
|  | 166.5-185.09 | 188.73-196.39 | 183.43-195.48 | 154.6-166.33 | 179.07-187.83 | 141.47-148.45 | 119.27-125.83 | 125.71-132.04 | 102.35-108.33 | 109.91-116.18 |
| **2006** | 173.20 | 197.78 | 192.41 | 174.70 | 185.01 | 135.87 | 127.37 | 134.38 | 106.81 | 121.85 |
|  | 163.9-182.5 | 193.83-201.73 | 186.09-198.73 | 168.42-180.98 | 180.59-189.44 | 132.43-139.32 | 124.02-130.71 | 131.12-137.65 | 103.76-109.86 | 118.6-125.1 |
| **2007** | 188.32 | 210.21 | 198.00 | 176.02 | 189.19 | 142.20 | 137.13 | 136.88 | 114.70 | 126.26 |
|  | 178.73-197.9 | 206.04-214.37 | 191.39-204.61 | 169.53-182.52 | 184.62-193.75 | 138.54-145.86 | 133.6-140.65 | 133.65-140.1 | 111.55-117.84 | 122.94-129.59 |
| **2008** | 206.01 | 221.58 | 215.92 | 185.92 | 202.87 | 152.24 | 145.86 | 149.19 | 116.67 | 141.00 |
|  | 195.69-216.33 | 217.24-225.93 | 208.29-223.55 | 178.92-192.93 | 198.08-207.66 | 148.3-156.18 | 142.18-149.54 | 145.8-152.58 | 113.49-119.86 | 137.43-144.58 |
| **2009** | 191.12 | 203.62 | 192.21 | 157.98 | 182.45 | 139.07 | 128.36 | 128.44 | 106.22 | 127.67 |
|  | 181.11-201.14 | 199.47-207.77 | 184.23-200.19 | 151.3-164.66 | 177.96-186.93 | 135.25-142.88 | 124.95-131.77 | 125.37-131.52 | 103.23-109.2 | 124.34-131 |
| **2010** | 217.42 | 224.76 | 191.29 | 164.59 | 200.78 | 150.10 | 138.89 | 139.18 | 109.42 | 136.14 |
|  | 206.39-228.44 | 220.26-229.25 | 183.14-199.45 | 156.3-172.87 | 195.98-205.58 | 145.95-154.24 | 135.26-142.52 | 135.96-142.4 | 106.4-112.43 | 132.65-139.64 |

|  | **IMD Quintile** | | | | |
| --- | --- | --- | --- | --- | --- |
|  | 0 (least deprived) | 1 | 2 | 3 | 4 (most deprived) |
| **Overall** | 111.42 | 121.05 | 133.93 | 151.46 | 206.08 |
| **95% CI** | 110.26-112.59 | 119.84-122.26 | 132.49-135.37 | 149.75-153.17 | 203.4-208.76 |
| **1997** | 86.67 | 92.23 | 98.29 | 114.58 | 156.35 |
|  | 83.82-89.52 | 89.49-94.96 | 95.21-101.38 | 111.09-118.07 | 151.51-161.19 |
| **1998** | 94.28 | 100.39 | 105.80 | 119.08 | 151.99 |
|  | 91.49-97.08 | 97.64-103.14 | 102.75-108.85 | 115.65-122.51 | 147.41-156.56 |
| **1999** | 100.27 | 100.28 | 105.90 | 122.08 | 166.95 |
|  | 97.62-102.92 | 97.68-102.88 | 103.03-108.78 | 118.75-125.41 | 162.21-171.7 |
| **2000** | 92.40 | 97.73 | 105.28 | 121.50 | 161.79 |
|  | 90-94.79 | 95.3-100.15 | 102.54-108.02 | 118.29-124.72 | 157.25-166.32 |
| **2001** | 99.28 | 106.67 | 119.49 | 127.18 | 167.42 |
|  | 96.81-101.75 | 104.16-109.18 | 116.57-122.41 | 123.95-130.41 | 162.83-172.01 |
| **2002** | 100.73 | 112.34 | 122.96 | 139.18 | 196.06 |
|  | 98.26-103.19 | 109.77-114.91 | 120-125.93 | 135.78-142.58 | 190.97-201.14 |
| **2003** | 114.15 | 122.98 | 137.64 | 159.96 | 218.27 |
|  | 111.49-116.81 | 120.27-125.69 | 134.45-140.83 | 156.22-163.69 | 212.76-223.78 |
| **2004** | 115.90 | 129.77 | 146.40 | 165.13 | 228.02 |
|  | 113.23-118.56 | 126.97-132.57 | 143.05-149.74 | 161.28-168.98 | 222.28-233.77 |
| **2005** | 118.15 | 128.83 | 146.47 | 167.33 | 227.71 |
|  | 115.45-120.84 | 126.05-131.61 | 143.1-149.85 | 163.41-171.25 | 221.89-233.52 |
| **2006** | 120.76 | 134.16 | 151.23 | 170.58 | 228.84 |
|  | 118.04-123.49 | 131.31-137.02 | 147.79-154.67 | 166.58-174.58 | 222.93-234.74 |
| **2007** | 121.56 | 136.08 | 157.38 | 175.22 | 242.34 |
|  | 118.84-124.28 | 133.2-138.95 | 153.86-160.9 | 171.14-179.3 | 236.17-248.51 |
| **2008** | 133.98 | 147.48 | 167.09 | 191.22 | 257.30 |
|  | 131.11-136.86 | 144.45-150.5 | 163.42-170.77 | 186.87-195.58 | 250.8-263.8 |
| **2009** | 117.72 | 130.41 | 150.12 | 174.76 | 248.70 |
|  | 115.09-120.34 | 127.62-133.2 | 146.68-153.56 | 170.6-178.91 | 242.21-255.2 |
| **2010** | 123.33 | 140.50 | 158.68 | 184.22 | 272.37 |
|  | 120.58-126.07 | 137.55-143.46 | 155.06-162.3 | 179.85-188.6 | 265.41-279.32 |

COPD – Chronic obstructive pulmonary disease

IMD – index of multiple deprivation
