# Supplementary material for: Incidence of Community-Acquired Lower Respiratory Tract Infections and Pneumonia among Older Adults in the United Kingdom: A Population-Based Study
Source: PLoS One. 2013 Sep 11;8(9):e75131. doi: 10.1371/journal.pone.0075131 (PMC3770598; doi:10.1371/journal.pone.0075131)
Supplement: Figure S1 — Age standardised incidence of LRTI including COPD exacerbation codes by sex over time. Standardised to UK population, mid-year 2004. (DOCX) [file pone.0075131.s004.docx]

**Figure S1. Age standardised incidence of LRTI including COPD exacerbation codes by sex over time.**
