# Supplementary material for: Incidence of Community-Acquired Lower Respiratory Tract Infections and Pneumonia among Older Adults in the United Kingdom: A Population-Based Study
Source: PLoS One. 2013 Sep 11;8(9):e75131. doi: 10.1371/journal.pone.0075131 (PMC3770598; doi:10.1371/journal.pone.0075131)
Supplement: Table S4 — Community-acquired pneumonia incidence rates overall and over time by sex, age, region and IMD quintile. (DOC) [file pone.0075131.s005.doc]

**Table S4. Community-acquired pneumonia incidence rates overall and over time by sex, age, region and IMD quintile.**

|  | **Overall** | **Sex** | | **Age (years)** | | | | | |
| --- | --- | --- | --- | --- | --- | --- | --- | --- | --- |
|  |  | Male | Female | 65-69 | 70-74 | 75-79 | 80-84 | 85-89 | ≥90 |
| **Overall** | 7.99 | 8.60 | 7.53 | 2.81 | 4.31 | 6.94 | 12.05 | 21.84 | 41.94 |
| **95% CI** | 7.92-8.07 | 8.49-8.72 | 7.44-7.62 | 2.74-2.87 | 4.22-4.4 | 6.81-7.08 | 11.82-12.27 | 21.39-22.29 | 40.87-43.01 |
| **1997** | 7.34 | 7.87 | 6.95 | 2.59 | 4.48 | 6.85 | 12.38 | 19.98 | 34.65 |
|  | 7.07-7.61 | 7.44-8.3 | 6.61-7.3 | 2.27-2.9 | 4.04-4.91 | 6.25-7.44 | 11.38-13.38 | 18.32-21.65 | 31.57-37.74 |
| **1998** | 7.69 | 7.67 | 7.71 | 2.88 | 4.30 | 7.31 | 12.98 | 20.98 | 35.54 |
|  | 7.43-7.95 | 7.26-8.07 | 7.36-8.05 | 2.56-3.19 | 3.9-4.71 | 6.74-7.89 | 11.98-13.98 | 19.36-22.6 | 32.6-38.48 |
| **1999** | 7.36 | 7.71 | 7.10 | 2.89 | 4.36 | 6.72 | 11.59 | 19.80 | 33.25 |
|  | 7.12-7.6 | 7.33-8.08 | 6.79-7.41 | 2.6-3.18 | 3.98-4.74 | 6.21-7.23 | 10.71-12.48 | 18.35-21.26 | 30.64-35.87 |
| **2000** | 6.30 | 6.70 | 6.00 | 2.16 | 3.40 | 6.11 | 9.37 | 17.70 | 27.31 |
|  | 6.09-6.51 | 6.37-7.03 | 5.73-6.27 | 1.92-2.39 | 3.09-3.71 | 5.65-6.58 | 8.65-10.09 | 16.4-19 | 25.09-29.52 |
| **2001** | 6.83 | 7.45 | 6.38 | 2.40 | 4.16 | 5.96 | 9.47 | 18.27 | 29.51 |
|  | 6.62-7.05 | 7.11-7.79 | 6.1-6.65 | 2.16-2.64 | 3.83-4.5 | 5.51-6.4 | 8.79-10.15 | 16.99-19.55 | 27.3-31.73 |
| **2002** | 6.92 | 7.48 | 6.50 | 2.43 | 3.88 | 6.43 | 9.90 | 17.48 | 27.95 |
|  | 6.71-7.14 | 7.14-7.81 | 6.23-6.78 | 2.2-2.66 | 3.56-4.2 | 5.97-6.89 | 9.23-10.57 | 16.24-18.73 | 25.85-30.04 |
| **2003** | 7.81 | 8.34 | 7.41 | 2.71 | 3.85 | 7.01 | 11.03 | 20.31 | 32.86 |
|  | 7.59-8.04 | 7.98-8.69 | 7.12-7.71 | 2.47-2.96 | 3.53-4.16 | 6.53-7.48 | 10.34-11.72 | 18.94-21.68 | 30.61-35.12 |
| **2004** | 7.93 | 8.33 | 7.62 | 2.68 | 4.23 | 7.05 | 11.10 | 18.42 | 33.16 |
|  | 7.7-8.16 | 7.98-8.69 | 7.32-7.91 | 2.45-2.92 | 3.9-4.56 | 6.58-7.52 | 10.42-11.78 | 17.13-19.71 | 30.91-35.41 |
| **2005** | 7.84 | 8.22 | 7.54 | 2.90 | 4.38 | 6.36 | 10.75 | 17.45 | 30.04 |
|  | 7.61-8.06 | 7.87-8.57 | 7.24-7.83 | 2.65-3.14 | 4.05-4.71 | 5.92-6.81 | 10.08-11.42 | 16.27-18.64 | 27.93-32.16 |
| **2006** | 8.16 | 8.75 | 7.70 | 2.87 | 4.38 | 6.73 | 11.35 | 16.57 | 30.57 |
|  | 7.93-8.39 | 8.38-9.11 | 7.4-8 | 2.63-3.12 | 4.05-4.71 | 6.27-7.19 | 10.65-12.04 | 15.46-17.68 | 28.44-32.69 |
| **2007** | 8.49 | 9.21 | 7.92 | 2.88 | 4.54 | 7.00 | 11.07 | 18.37 | 30.74 |
|  | 8.26-8.73 | 8.84-9.59 | 7.62-8.23 | 2.64-3.12 | 4.21-4.87 | 6.54-7.47 | 10.38-11.75 | 17.22-19.52 | 28.59-32.89 |
| **2008** | 9.91 | 10.66 | 9.30 | 3.57 | 5.03 | 7.77 | 13.41 | 20.92 | 36.12 |
|  | 9.64-10.17 | 10.25-11.07 | 8.96-9.64 | 3.3-3.85 | 4.67-5.38 | 7.28-8.27 | 12.64-14.18 | 19.7-22.15 | 33.73-38.52 |
| **2009** | 9.52 | 10.44 | 8.77 | 3.18 | 5.00 | 7.44 | 12.81 | 20.28 | 34.84 |
|  | 9.26-9.78 | 10.03-10.84 | 8.44-9.1 | 2.93-3.44 | 4.64-5.35 | 6.95-7.92 | 12.05-13.57 | 19.08-21.48 | 32.48-37.19 |
| **2010** | 10.06 | 10.82 | 9.44 | 3.61 | 4.87 | 7.84 | 13.37 | 21.49 | 34.97 |
|  | 9.79-10.33 | 10.4-11.24 | 9.09-9.79 | 3.34-3.88 | 4.52-5.23 | 7.33-8.35 | 12.59-14.16 | 20.23-22.76 | 32.69-37.25 |

|  | **Region** | | | | | | | | | |
| --- | --- | --- | --- | --- | --- | --- | --- | --- | --- | --- |
|  | North East | North West | Yorkshire & The Humber | East Midlands | West Midlands | East of England | South West | South Central | London | South East Coast |
| **Overall** | 10.21 | 9.11 | 8.73 | 7.93 | 8.71 | 8.25 | 8.77 | 10.27 | 7.76 | 7.18 |
|  | 9.61-10.81 | 8.88-9.33 | 8.37-9.09 | 7.58-8.28 | 8.45-8.96 | 8.01-8.48 | 8.51-9.02 | 10.01-10.54 | 7.53-8 | 6.95-7.4 |
| **1997** | 7.35 | 7.27 | 7.64 | 6.87 | 6.69 | 7.41 | 7.65 | 10.99 | 7.73 | 6.72 |
|  | 5.8-8.9 | 6.55-7.99 | 6.53-8.74 | 5.73-8 | 5.87-7.5 | 6.6-8.22 | 6.7-8.6 | 9.79-12.18 | 6.83-8.64 | 5.83-7.62 |
| **1998** | 7.38 | 7.50 | 7.69 | 7.31 | 7.78 | 8.90 | 7.57 | 9.79 | 7.75 | 7.57 |
|  | 5.79-8.96 | 6.79-8.21 | 6.61-8.77 | 6.19-8.42 | 6.95-8.61 | 8.05-9.74 | 6.7-8.43 | 8.77-10.8 | 6.87-8.64 | 6.63-8.51 |
| **1999** | 8.95 | 6.65 | 7.33 | 6.89 | 7.46 | 7.54 | 8.89 | 9.71 | 6.56 | 6.72 |
|  | 7.18-10.72 | 6-7.29 | 6.29-8.38 | 5.87-7.9 | 6.67-8.24 | 6.81-8.28 | 8.05-9.73 | 8.82-10.6 | 5.81-7.31 | 5.91-7.54 |
| **2000** | 8.15 | 6.21 | 6.81 | 5.72 | 6.98 | 6.59 | 5.98 | 7.77 | 7.29 | 4.61 |
|  | 6.52-9.78 | 5.6-6.81 | 5.82-7.8 | 4.78-6.65 | 6.27-7.7 | 5.92-7.25 | 5.31-6.64 | 7.06-8.49 | 6.55-8.04 | 3.98-5.24 |
| **2001** | 8.05 | 7.31 | 7.79 | 6.24 | 7.47 | 7.63 | 7.23 | 8.28 | 7.78 | 5.02 |
|  | 6.4-9.7 | 6.67-7.95 | 6.74-8.83 | 5.28-7.19 | 6.72-8.21 | 6.93-8.34 | 6.49-7.96 | 7.57-8.99 | 7.03-8.53 | 4.38-5.66 |
| **2002** | 8.68 | 8.24 | 7.63 | 6.93 | 8.09 | 6.94 | 7.13 | 8.68 | 6.78 | 5.07 |
|  | 6.94-10.43 | 7.57-8.9 | 6.62-8.64 | 5.92-7.94 | 7.31-8.87 | 6.28-7.6 | 6.41-7.86 | 7.94-9.41 | 6.09-7.46 | 4.45-5.7 |
| **2003** | 10.89 | 8.83 | 8.56 | 7.66 | 9.10 | 7.63 | 7.64 | 11.57 | 7.03 | 6.04 |
|  | 8.89-12.88 | 8.13-9.52 | 7.46-9.67 | 6.59-8.73 | 8.27-9.93 | 6.94-8.33 | 6.9-8.38 | 10.71-12.44 | 6.32-7.74 | 5.37-6.71 |
| **2004** | 9.81 | 9.59 | 9.55 | 8.59 | 9.25 | 7.17 | 8.56 | 10.52 | 7.79 | 5.93 |
|  | 7.91-11.71 | 8.86-10.32 | 8.37-10.74 | 7.42-9.76 | 8.41-10.1 | 6.5-7.84 | 7.78-9.34 | 9.69-11.34 | 7.03-8.55 | 5.28-6.59 |
| **2005** | 13.10 | 9.44 | 8.95 | 7.61 | 9.45 | 7.87 | 8.41 | 9.73 | 5.98 | 7.63 |
|  | 10.87-15.34 | 8.71-10.16 | 7.79-10.1 | 6.5-8.72 | 8.58-10.32 | 7.16-8.58 | 7.64-9.18 | 8.94-10.53 | 5.33-6.64 | 6.88-8.38 |
| **2006** | 12.13 | 9.76 | 8.87 | 8.81 | 8.74 | 8.03 | 8.84 | 10.88 | 6.68 | 7.75 |
|  | 9.96-14.29 | 9-10.51 | 7.67-10.06 | 7.59-10.03 | 7.91-9.58 | 7.29-8.77 | 8.06-9.63 | 10.03-11.73 | 5.97-7.38 | 7-8.49 |
| **2007** | 10.73 | 10.58 | 11.14 | 8.91 | 9.35 | 8.12 | 10.16 | 10.72 | 7.72 | 7.64 |
|  | 8.74-12.72 | 9.78-11.37 | 9.73-12.54 | 7.64-10.17 | 8.47-10.23 | 7.35-8.9 | 9.31-11.01 | 9.9-11.54 | 6.97-8.47 | 6.89-8.38 |
| **2008** | 14.17 | 12.12 | 11.19 | 10.36 | 11.18 | 10.29 | 11.23 | 13.36 | 9.39 | 10.18 |
|  | 11.81-16.53 | 11.26-12.99 | 9.64-12.74 | 8.92-11.8 | 10.21-12.15 | 9.38-11.19 | 10.33-12.13 | 12.44-14.28 | 8.56-10.22 | 9.31-11.06 |
| **2009** | 12.14 | 11.68 | 12.99 | 11.71 | 10.02 | 11.23 | 10.26 | 10.85 | 10.11 | 10.42 |
|  | 9.93-14.35 | 10.82-12.53 | 11.11-14.86 | 10.1-13.33 | 9.1-10.94 | 10.26-12.2 | 9.4-11.11 | 10.03-11.67 | 9.25-10.97 | 9.54-11.3 |
| **2010** | 14.53 | 12.84 | 12.88 | 12.74 | 10.94 | 12.26 | 11.00 | 11.46 | 10.06 | 10.68 |
|  | 12.06-17 | 11.93-13.76 | 10.96-14.79 | 10.7-14.78 | 9.96-11.91 | 11.21-13.32 | 10.1-11.91 | 10.62-12.3 | 9.2-10.91 | 9.77-11.59 |

|  | **IMD Quintile** | | | | |
| --- | --- | --- | --- | --- | --- |
|  | 0 (least deprived) | 1 | 2 | 3 | 4 (most deprived) |
| **Overall** | 7.58 | 8.35 | 9.93 | 10.08 | 12.78 |
|  | 7.38-7.77 | 8.15-8.55 | 9.68-10.17 | 9.82-10.35 | 12.4-13.15 |
| **1997** | 7.27 | 6.63 | 7.72 | 7.71 | 8.46 |
|  | 6.47-8.06 | 5.93-7.33 | 6.9-8.54 | 6.86-8.56 | 7.45-9.47 |
| **1998** | 6.89 | 7.49 | 8.80 | 8.54 | 10.47 |
|  | 6.17-7.61 | 6.78-8.2 | 7.96-9.63 | 7.68-9.4 | 9.37-11.57 |
| **1999** | 7.48 | 6.84 | 8.23 | 8.55 | 9.45 |
|  | 6.8-8.17 | 6.2-7.48 | 7.48-8.98 | 7.74-9.37 | 8.45-10.46 |
| **2000** | 5.61 | 6.10 | 7.32 | 7.53 | 8.87 |
|  | 5.06-6.17 | 5.54-6.67 | 6.65-7.99 | 6.81-8.26 | 7.93-9.81 |
| **2001** | 6.45 | 6.88 | 8.66 | 7.69 | 9.94 |
|  | 5.86-7.04 | 6.3-7.47 | 7.94-9.38 | 6.98-8.4 | 8.96-10.92 |
| **2002** | 6.61 | 7.15 | 8.28 | 8.43 | 10.82 |
|  | 6.03-7.2 | 6.56-7.74 | 7.58-8.98 | 7.69-9.17 | 9.8-11.85 |
| **2003** | 7.59 | 7.65 | 10.15 | 9.08 | 11.91 |
|  | 6.96-8.22 | 7.05-8.26 | 9.37-10.94 | 8.31-9.86 | 10.82-13.01 |
| **2004** | 7.71 | 8.41 | 10.63 | 8.92 | 12.79 |
|  | 7.08-8.34 | 7.77-9.04 | 9.81-11.44 | 8.14-9.69 | 11.64-13.95 |
| **2005** | 7.20 | 8.58 | 9.76 | 10.20 | 13.61 |
|  | 6.59-7.81 | 7.94-9.23 | 8.98-10.54 | 9.36-11.04 | 12.39-14.84 |
| **2006** | 7.99 | 8.48 | 10.14 | 10.81 | 13.50 |
|  | 7.35-8.63 | 7.84-9.11 | 9.34-10.93 | 9.93-11.68 | 12.26-14.74 |
| **2007** | 8.14 | 9.13 | 10.83 | 12.01 | 15.83 |
|  | 7.49-8.78 | 8.46-9.79 | 10-11.65 | 11.08-12.94 | 14.47-17.2 |
| **2008** | 9.65 | 10.62 | 13.28 | 14.35 | 17.82 |
|  | 8.94-10.35 | 9.9-11.34 | 12.35-14.2 | 13.32-15.38 | 16.34-19.31 |
| **2009** | 9.67 | 10.62 | 12.61 | 13.24 | 18.48 |
|  | 8.97-10.37 | 9.91-11.34 | 11.71-13.51 | 12.24-14.24 | 16.92-20.04 |
| **2010** | 9.29 | 11.33 | 13.31 | 14.11 | 20.35 |
|  | 8.59-9.99 | 10.57-12.08 | 12.37-14.26 | 13.05-15.17 | 18.68-22.01 |

IMD – index of multiple deprivation
